# Why evolution matters for species conservation: perspectives from three case studies of plant metapopulations

**DOI:** 10.1111/eva.12336

**Published:** 2015-11-19

**Authors:** Isabelle Olivieri, Jeanne Tonnabel, Ophélie Ronce, Agnès Mignot

**Affiliations:** ^1^ Institut des Sciences de l'EvolutionUniversité MontpellierCNRSIRDEPHECC65Place Eugène Bataillon, 34095, Montpellier cedex 5France; ^2^ Department of Ecology and EvolutionLe BiophoreUNIL‐SORGEUniversity of LausanneLausanneSwitzerland

**Keywords:** conservation genetics, contemporary evolution, dispersal, management, mating systems, natural selection, phylogenetics

## Abstract

We advocate the advantage of an evolutionary approach to conservation biology that considers evolutionary history at various levels of biological organization. We review work on three separate plant taxa, spanning from one to multiple decades, illustrating extremes in metapopulation functioning. We show how the rare endemics *Centaurea corymbosa* (Clape Massif, France) and *Brassica insularis* in Corsica (France) may be caught in an evolutionary trap: disruption of metapopulation functioning due to lack of colonization of new sites may have counterselected traits such as dispersal ability or self‐compatibility, making these species particularly vulnerable to any disturbance. The third case study concerns the evolution of life history strategies in the highly diverse genus *Leucadendron* of the South African fynbos. There, fire disturbance and the recolonization phase after fires are so integral to the functioning of populations that recruitment of new individuals is conditioned by fire. We show how past adaptation to different fire regimes and climatic constraints make species with different life history syndromes more or less vulnerable to global changes. These different case studies suggest that management strategies should promote evolutionary potential and evolutionary processes to better protect extant biodiversity and biodiversification.

## Introduction

This study belongs to a special issue on women's contribution to evolutionary biology. Through the review of past work of our group, led by Isabelle Olivieri (Box 1), we seek to illustrate why evolution needs to be considered in conservation biology, based on extensive studies of the recent evolutionary history in three plant taxa. Conservation biology should not only consist in conserving habitats, species richness or species, but it should be about promoting evolutionary potential and evolutionary processes at all levels of biodiversity (Box 2). We first briefly review general ideas about why conservation biology benefits from an evolutionary perspective, and then introduce three case studies that will illustrate how our work contributed to this debate.

Box 1 **Personal reflections.**

Our group started its investigations in evolutionary conservation biology in 1993. Conservation biology then largely ignored evolutionary considerations. At best, in the nineties, population viability analyses included inbreeding depression (Lacy 1993). Genetic studies were rare, and then, only neutral molecular diversity was considered (Allendorf and Servheen 1986), often at a single point in time (but see Noss 1990). A new concept was then gaining much influence on the paradigms of conservation biology, that of metapopulations. In 1983, while IO was doing a postdoc at Stanford University, metapopulation was a big word in conservation biology, but studied from the demographic point of view only (Harrison et al. 1988). There was at that time no evolution being considered. It was around that time that IO initiated a new line of research investigating how a metapopulation functioning affects the evolution of dispersal and other life history traits, joined by several students and collaborators (among which OR) throughout the years (e.g. Olivieri and Gouyon 1985, 1997; Olivieri et al. 1990, 1995; Gandon et al. 1996; Ronce and Olivieri 1997; Godelle et al. 1998; Brachet et al. 1999; Ronce et al. 2000a,b; Olivieri 2000, 2001; Mathias et al. 2001; Ravigné et al. 2004; Ravigné et al. 2006; Ravigné et al. 2009; Vitalis et al. 2004, 2014). This research was in large part theoretical, but also included experimental studies of dispersal in thistles and of plant genetics, much inspired by the metapopulation concept (Manicacci et al. 1992; Colas et al. 2001; Vitalis et al. 2001, 2002). Several opportunities allowed our group to initiate what were then pioneering studies in evolutionary conservation biology with a metapopulation perspective.

Opportunity to work on *Centaurea corymbosa* occurred when IO was asked to study some endemic rare species of the Languedoc–Roussillon region by Louis Olivier, then Head of the National Botanical Mediterranean Conservatory of Porquerolles (CBNMP), a conservation structure in Southern France. At that time, she was asked to perform an allozyme study for five endemics of the region. Instead, thanks to the work of Bruno Colas who started a PhD, she focused on only one of them, *C. corymbosa*, and, joined by Miquel Riba, they started a programme on plant evolutionary conservation biology. They aimed at understanding rarity in this species. This question was further explored by several other PhDs and postdoctorates within and then outside the group as former students continued working on this fascinating model.

Opportunity to work on *Brassica insularis* was similarly due to rather contingent circumstances. IO was asked by the French Ministry of Research to work on a protected Corsican species, which might be a useful genetic resource. Studies on *B*. *insularis*, which our group initiated in 1998, thus allowed us to examine the generality of patterns found in *C. corymbosa*, and to examine further some questions that arose in our study of *C. corymbosa* due to the availability of genetic resources in *Brassica*. First, collections of plants from different populations maintained *ex situ* by the CBNMP were available for us to measure traits in controlled conditions. Second, close relatedness to the domestic cabbage allowed us to use genetic resources, which were not available for nonmodel species such as *C. corymbosa*, and in particular to study the genetics of self‐incompatibility*, which was of particular interest for AM.

In 1999, IO was asked by the Centre National de la Recherche Scientifique (CNRS) to organize a Biodiversity workshop in South Africa, where apartheid had ceased and the French government wanted to seed new scientific collaborations. IO and AM participated at the workshop, where contacts were initiated with South African researchers (in particular Jeremy Midgley, Anthony Rebelo, William Bond, with whom the collaboration went further) on the evolution of diversity and life histories in the fynbos. The PhD of JT allowed these projects to make a significant step forward in recent years.

These biographical elements illustrate how exciting science can be born from fortuitous meetings and circumstances, how solicitations from conservation structure and political powers can seed rich long‐term research programme with high interest both for basic and applied science. Most of all, it illustrates that good science emerges from cooperation of many students, young and experienced researchers. This research was the achievement of a group with strong bonds, complementary skills and high diversity. Working in this group was scientifically, but also personally, enlightening. This is our experience of research, as female scientists at various stages of their career: we feel very lucky having worked in an environment where cooperation was always valued above competition, and where friendship was intricately mixed with high intellectual stimulation.

Box 2 **Conservation of biodiversity beyond species.**

More than 300 years ago, Carl Linnaeus offered a system of classification (Linnæus 1735). Species played a central role, and were viewed as fixed entities created by God. A hundred and fifty years later, Charles Darwin discovered a major evolutionary process (selection) and suggested a new way of classifying living organisms. Darwin wrote that *the characters which naturalists consider as showing true affinity between any two or more species, are those which have been inherited from a common parent, and, in so far, all true classification is genealogical* (Darwin 1859). It was then clear that species were not to be defined on the basis of what they did, but on the basis of what they were and where they came from (Lecointre 2011; Jetz et al. 2014). Darwin wrote *I am often in despair in making the generality of naturalists even comprehend me. Intelligent men who are not naturalists and have not a bigoted idea of the term species, show more clearness of mind* (Darwin 1859). Species exist, just as other levels of classification, because we invented them. They are one arbitrary level in the Tree of life. There is often as much diversity within a species as between species (Helms et al. 2015; Realini et al. 2015), so that one could have cut the Tree of life elsewhere to define taxonomical units to be preserved.

Evolution and ecology can operate on the same time scale. There are many examples of rapid contemporary evolution in nature (e.g. Hansen et al. 2012; see special issue introduced by Merilä and Hendry 2014) and in experimentally evolving populations (Kawecki et al. 2012; Rosenzweig and Sherlock 2014). Evolutionary conservation biology emerged recently as a new discipline (Ferrière et al. 2004; Whiteley et al. 2015), in part because of the growing awareness that evolutionary changes can proceed sufficiently fast to affect demographic functioning. Understanding the threats on population viability requires considering not only population sizes but also the genetic diversity of populations. This idea was early recognized by studies of impacts of inbreeding depression on population viability, now enriched by our increasing understanding of the feedbacks between demography and genetics (Keller and Waller 2002; Cheptou and Donohue 2011). Preserving genetic diversity among populations, in particular that linked to local adaptations, implies understanding the evolutionary forces that have shaped this diversity (Hansen et al. 2012; Santamaria and Mendez 2012). The development of genomics offers new insights into answering these questions (Ouborg 2010; Thomson et al. 2010; Angeloni et al. 2012; Mc Mahon et al. 2014; but see Shaffer et al. 2015). Awareness about forecasted increases in population extinction and species range contraction has renewed interest on the concept of evolutionary rescue (see special issue introduced by Gonzalez et al. 2013; see also Orr and Unckless 2014), a process by which a declining population evolves adaptation to new stressful conditions and escapes extinction. Threatened lineages need to evolve to persist in the context of global changes, but often have small population sizes and evolutionary histories that constrain their ability to adapt to new conditions, making evolutionary rescue particularly unlikely (van der Wal et al. 2013). Past evolution can indeed explain the vulnerability of species to threats (Corey and Waite 2008; Baker et al. 2010; van Woesik et al. 2012; Lavergne et al. 2013).

The contribution of our group (Box 1) to the development of the field of evolutionary conservation biology was much inspired by the concept of metapopulations. The term metapopulation was first coined by Levins (1968) in the context of island biogeography (‘a population of local populations which are established by colonists, survive for a while, send out migrants, and eventually disappear’), then by Slatkin and Wade (1978) in the context of population genetics, and initially used in conservation biology as a pure demographic tool by Hanski (1989) (see Couvet et al. 1985; Olivieri et al. 1990 for reviews). We will illustrate, through three case studies, how concepts about evolution in metapopulations may allow us to understand the vulnerability of different populations, and propose management strategies to better protect populations and their diversity of ecological traits. In return, these investigations enriched and stimulated our conceptual understanding of evolutionary biology in metapopulations. These different case studies represent some extremes in metapopulation functioning.

The first case study concerns the biology of the rare endemic *Centaurea corymbosa*, which has an extremely restricted range (see Box 3 for a description about the biology and natural history of this species). It illustrates the conservation issues associated with sets of populations that do not function as metapopulations but still live in patchy habitats, that is where dispersal between extant populations and colonization of new patches are absent, and where founding of new populations cannot balance potential local extinctions. Our research suggested how evolution of dispersal, mating system and life history traits in the landscape inhabited by C. *corymbosa* may have worsened its persistence prospects in the long term, a potential concrete case of evolutionary suicide. The same types of challenges associated with the combination of strong isolation and small population sizes were illustrated by our research on another narrow endemic with a highly fragmented habitat, *Brassica insularis* (Box 4). The third case study, discussed in the second part of this review, reflects the opposite extreme in metapopulation functioning and concerns the evolution of fire‐related traits, and more generally of life history strategies, in the highly diverse genus *Leucadendron* (Box 5) of the South African fynbos, a world hot spot of biodiversity. There, fire disturbance and the recolonization phase after fires are so integral to the functioning of populations that the recruitment of new individuals in the populations is conditioned by the occurrence of fire. Combining theory and comparative analyses based on phylogenies, our results suggest that the large diversity of life history traits has evolved jointly with fire regimes and climatic conditions. Examining evolutionary history, we are able to predict which traits are currently most threatened by changes in climate and fire regimes. Finally, we discuss how an evolutionary perspective could affect management in these different case studies and conclude by defining a research agenda for evolutionary conservation biology.

Box 3 ***Centaurea corymbosa,* a cliff‐dwelling species tottering on the brink of extinction.**

Our work on *Centaurea corymbosa* has become a model in conservation biology (e.g. Wilson and Rannala 2003). The species, from the family *Asteraceae*, is endemic of the Clape Massif (France). Only six populations of *C. corymbosa* have been described since 1783. They all are on the south‐western slope of the Massif, occupying less than 10% of the area. The species range is thus limited to three km^2^. Populations are restricted to cliffs and the edge of cliffs, on rocky and open areas with very shallow soils. The species appears as a poor competitor, restricted to open areas with very little vegetation cover (Imbert et al. 2012). Distance between populations varies from 300 to 2300 m, but populations are separated by what represents a hostile environment for *C. corymbosa* (Colas et al. 1997). *Centaurea corymbosa* is a monocarpic* plant: it flowers after several years of vegetative growth as a rosette (between 3 and 12 years, with a mean of 5.5 years; Fréville et al. 2004). *Centaurea corymbosa* is a self‐incompatible and insect‐pollinated plant (Kirchner et al. 2005).

The species is thus very rare and, as such, endangered. It is listed under Bern Convention and under Annex II of EC Habitats Directive, as well as under the list of plants protected in France (Ministerial Order of 20 January, 1982, published 13 May, 1982), and under Volume I (Priority Species) of the National Red List of Threatened Plants in France (Olivier et al. 1995).

Box 4 ***Brassica insularis,* another endangered cliff species.**

The insular cabbage *Brassica insularis,* which is closely related to the cultivated cabbage, *Brassica oleracea,* is considered as vulnerable at the national and international levels, and listed under different nature protection mechanisms. As *C. corymbosa*,* B. insularis* is an endemic species, found mainly in some Mediterranean Isles (Corsica, Sardinia, Pantelleria) and also in Tunisia and Algeria (Snogerup et al. 1990). Its habitat is restricted to limestone cliffs, and it occurs in isolated populations of various sizes. As *C. corymbosa* and *B*. *oleracea*,* B. insularis* is a perennial*, self‐incompatible plant, pollinated by insects (Snogerup et al. 1990). It is however polycarpic* with a life span of 5–7 years and an age at first reproduction of 2 or 3 years. The strong morphological differentiation between Corsican populations has led to the distinction of six varieties (Widler and Bocquet 1979), five of them existing only in one population.

Box 5 Life history traits of perennial* plants in the South African fynbos, the special case of the genus *Leucadendron*.

The genus *Leucadendron*, or Conebushes, belongs to the Proteaceae family, one of the most prominent flowering plant families in the southern hemisphere. This genus is mostly distributed in a restricted area, the South African fynbos, known to display a large environmental variation both for climate and for fire regimes (Schulze 1997). Moreover, environments inhabited by each *Leucadendron* species are very well characterized (Rebelo 1992). All 96 taxa in the genus are dioecious* shrubs. Some species show no morphological differences between males and females, whereas other species exhibit the highest degrees of sexual dimorphism in angiosperms. Once mature, female plants produce every year flowerheads that form woody cones in which fruits are borne. Species are either serotinous, retaining seeds in cones for several years, or display a soil‐stored seed bank*. Fire triggers seed recruitment: in serotinous species, it leads to seed release from cones, and in soil‐stored seed bank* species, it breaks seed dormancy. In this genus as in many other *Proteaceae*, keeping cones closed requires providing them with some resources, mainly water. Death of the plants results in premature opening of the cones and in seed release under unfavourable conditions for recruitment. Some species are able to regrow from protected buds after a fire: these resprouting* individuals will thus experience several fires. On the contrary, individuals of non‐resprouting* species are killed by fire events. In the genus *Leucadendron*, these fire‐related traits are found in different species: 45.7% and 8.6% of species are, respectively, serotinous non‐resprouting* and serotinous resprouting*, while 42.0% and 3.7% display underground‐stored seed banks and are, respectively, non‐resprouting* and resprouting*. No species is known to have both a serotinous and a soil‐stored seed bank*. Pollination mode varies between *Leucadendron* species: pollen can be dispersed either by wind or by insects. When seeds are released from the cones, they can be dispersed by wind, mammals, ants or gravity.

## Part 1: Are rare species caught in an evolutionary trap?

### Rarity and extinction vortex

Species rarity may be defined according to population size, geographical range and habitat range: there is a single way to be common (i.e. large populations, large distribution and wide range of habitats), but there are seven forms of rarity (Rabinowitz 1981). We studied two rare species, *C. corymbosa* (Box 3) and *B. insularis* (Box 4), both characterized by a narrow distribution and small populations (with some exceptions for *B. insularis*). We performed studies coupling genetics, demography, ecology and reproduction biology to identify the causes of rarity on one hand, and to be able to provide pragmatic solutions on the other hand. Very small populations can be caught in an extinction vortex, where demographic vulnerability is accentuated by the lack of genetic diversity, inbreeding depression and fixation load, leading to even smaller population sizes and greater genetic problems (Oostermeijer et al. 2003; Frankham 2005; Fagan and Holmes 2006; Wagenius et al. 2007; Leducq et al. 2010). Life history traits of *C. corymbosa* and *B. insularis* further increase their demographic vulnerability. Evolutionary rescue through the evolution of different life history traits, which could boost population dynamics, is prevented not only by the lack of genetic variability associated with small population size, but also by selection pressures associated with demographic functioning and habitat structure (Colas et al. 1997; Noel et al. 2010).

### *Centaurea corymbosa*: demographic threats

*Centaurea corymbosa* has an extremely narrow range, with only six populations (Box 3). Demographic surveys have revealed that populations are declining (Colas et al. 1996, 1997; Riba et al. 2001). Population size (number of flowering plants) varies among populations and years (e.g. between one and 36 individuals *per* year in the smallest population, and between 76 and 351 in the largest). Demographic surveys in permanent quadrats performed every 3 months allowed the estimation of transition rates between different stages of the life cycle, and the modelling of population dynamics using matrix models to predict long‐term population growth rates (Fréville et al. 2004). Based on modelling results, we predict decline in three populations of the six known populations. The predicted growth rates however vary among populations and years. Demographic analyses have revealed that demographic stochasticity, due to small population size, and environmental stochasticity, due to varying environmental conditions in space and time, explain this high variability in population growth (Fréville et al. 2004). We predicted extinction for at least two populations within the next 100 years (Fréville 2001).

### An evolutionary hypothesis: Fragmentation of favourable habitat and associated dispersal cost has counterselected traits favouring dispersal.

The habitat of *C. corymbosa* is naturally highly fragmented, with rare patches of favourable habitat on rocky cliffs, surrounded by vast areas where germination and life cycle completion is impossible. The degree of fragmentation is also probably influenced by human activities in a complex manner (see Part 3). Dispersal of seeds in such a landscape is an extremely risky venture, with a very small probability of landing in a suitable patch of habitat. Theoretical studies have predicted that a high cost of dispersal in such fragmented landscapes can counterselect traits enhancing seed dispersal distances (e.g. van Valen 1971; Olivieri et al. 1995; see a review in Ronce 2007). Empirical support for this theoretical prediction has been found in two other *Asteraceae* species (Cheptou et al. 2008; Riba et al. 2009), showing that the consequences of habitat fragmentation can be aggravated by the evolution of dispersal for species remaining in these fragments.

### Dispersal is indeed very limited in *C. corymbosa*

Fruits of *C. corymbosa* bear a pappus* (see glossary in Box 6), which is usually characteristic of adaptation to seed dispersal by wind in *Asteraceae*. The peculiar morphology of the pappus* in *C. corymbosa* makes seed dispersal by wind very inefficient: falling velocity of fruits, which is inversely related to seed dispersal distance under a given height of release, is four times higher than for dandelions fruits (Riba et al. 2005). Dispersal thus occurs mostly by gravity, which is consistent with the estimation of very short average dispersal distances, as measured by *in situ* direct methods (*d* = 32 cm, Colas et al. 1997). Additionally, there is no evidence that the elaiosome* facilitates dispersal by ants (Imbert 2006).

Box 6 **Glossary.**

**Dioecious:** species with distinct male and female plants.

**Elaiosome**: an external structure that belongs to a seed or a fruit, rich in nutrients, and that is attractive to ants.

**Monocarpic:** a plant that produces seeds only once before dying.

**Pappus:** a parachute‐like structure on fruits of *Asteraceae* that facilitates dispersal by wind.

**Polycarpic:** a plant that flowers and sets seeds several times in its lifetime.

**Perennial:** a plant that lives more than one year. Monocarpy and polycarpy are two different strategies that exist in perennial plants.

**Resprouting:** the ability of an organism to regrow from burgeons after that aerial parts were killed, e.g. after a fire.

**Self‐incompatibility:** inability for a fertile hermaphrodite plant to produce viable seeds by self‐pollination. There are different genetic self‐incompatibility systems, but they all exhibit a large number of alleles at each locus, called **S‐alleles**, maintained by frequency‐dependent selection. An S‐allele cannot fertilize the same S‐allele.

**Serotiny**: the retention of seeds within closed cones in the plant canopy after seed maturation. Seeds are normally released after fire (i.e. when cones die) and are short‐lived after release. They germinate with the first following rains.

**Soil‐stored seed bank:** dormant seeds are released every year and stored in the soil. In the specific case of species living in fire‐prone environment, seeds generally germinate when a fire occurs.

In the context of the highly fragmented structure of the habitat, this very limited dispersal capacity of seeds explains the demographic isolation of the different populations, which function as independent entities. Fine‐scale analysis of spatial patterns of gene flow through pollen within the Auzils population has shown that the average dispersal distance of pollen was lower than 22 m, and has revealed the quasi‐absence of immigrant pollen from other populations (Hardy et al. 2004a). Together with limited pollen dispersal, short seed dispersal distance thus contributes to the genetic isolation and large genetic distance among populations despite the small geographical distances between them (*F*_st_ = 0.23 for microsatellites and 0.35 for allozymes; Fréville et al. 2000, 2001). Genetic assignation tests also revealed that immigrants within populations were rare (209 of 228 individuals were assigned to their own population) and came from populations within a distance of 300–600 m (Fréville et al. 2001).

### The range of *C. corymbosa* is limited by its poor dispersal capacity

During our 20‐year demographic survey, we recorded no new populations in the area. Five of the six populations of *C. corymbosa* have been known for more than a century, and even if the sixth population was discovered in 1994, it may well have existed before that. The absence of colonization of new sites could be due to dispersal limitation or to the absence of favourable patches of habitats. We tested the latter hypothesis by introducing seeds of *C. corymbosa* in two new sites. We found that plants had a better survival in those new sites than in the previously occupied sites (probably because we established the seeds in what we evaluated as favourable microsites), but had a lower fecundity (probably because of Allee effects due to the small number of introduced plants, Colas et al. 2008). This experiment should be repeated, in particular because one of the introduced populations went extinct in 2014, but it however showed unambiguously that empty favourable sites do exist in the area, where plants can survive, but cannot be reached by *C. corymbosa* due to its poor dispersal capacity.

### The absence of colonizations has many evolutionary consequences

The combination of perenniality, monocarpy and self‐incompatibility* (which are traits shared by closely related species in the genus *Centaurea*, see Fréville et al. 1998) makes the populations of *C. corymbosa* particularly vulnerable to any disturbance that would strongly decrease population density, resulting in strong Allee effects. The late age at first reproduction slows down population growth, and successful reproduction requires finding synchronous and compatible partners, which could be really challenging in a small population, as exemplified by our introduction attempts. Paternity analyses in the Auzils population showed that seeds from a single plant were sired on average by five different pollen donors only (Hardy et al. 2004b). We further found that seed set increased with the density of neighbouring plants, and showed that it was likely due to the self‐incompatibility* system (Kirchner et al. 2005). Self‐compatibility has evolved many times from self‐incompatibility* in angiosperms (Igic et al. 2003). Self‐pollination experiments have shown that a few individuals of *C. corymbosa* are partially self‐compatible (10–15%) or fully self‐compatible (1%, unpublished results). So why did the populations not evolve greater self‐compatibility given the threats on persistence that such a reproductive system entails in small, declining populations? Similar questions arise about the evolution of lower flowering age and iteroparity.

**Figure 1:**
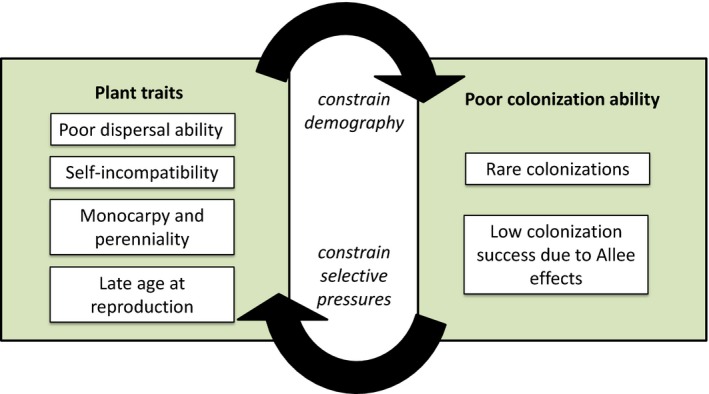
The extinction vortex of *C. corymbosa*. The plant traits, because they act on demography (arrow from left to right, top), induce a poor colonizing ability, which in return (other arrow) determines plant traits.

Theoretical studies on the evolution of life history traits and mating systems (reviewed in Ronce and Olivieri 2004) shed some light on these questions. In a metapopulation, selection pressures on mating and life history strategies differ during phases of recolonization after disturbance, where populations are small but growing, from selection pressures in stable populations. As a result, organisms living in metapopulations with a higher population turnover have been predicted to evolve lower age at maturity (de Jong et al. 2000), higher selfing rates (Pannell and Barrett 2001), higher fecundities (Ronce and Olivieri 1997; Crowley and McLetchie 2002) and higher dispersal ability (e.g. Olivieri et al. 1995). In *C. corymbosa*, such a metapopulation functioning is disrupted: in the absence of colonization, there is simply no selection pressures favouring other life history and mating strategies. If a rare colonization occurred, the present combination of traits would result in large Allee effects, making it even less likely that such a colonization would succeed and establish a viable population. Theoretical studies have shown how such evolutionary traps could ultimately lead to extinction, a process called ‘evolutionary suicide’ (reviewed in Ferrière and Legendre 2013). *Centaurea corymbosa* (Fig. 1) could well be a concrete example of a species on the verge of such evolutionary suicide (Ferrière et al. 2004), with all populations in decline and short‐term extinction predicted for two of six. Many other species that evolved life history traits that increase their demographic vulnerability may be in the same situation, but the combination of traits increasing extinction risk will depend on environmental pressures that the species confront (see e.g., Fréville et al. 2007 for experimental results on grassland communities).

### *Brassica insularis*: another endemic rare cliff species with similar conservation challenges

Do the demographic and evolutionary challenges faced by *C. corymbosa* represent an isolated and even pathological case, or could our understanding of these challenges inform the conservation biology of other species facing similar challenges? Studies on *B. insularis*, which shares many characteristics of its biology and demography with *C. corymbosa,* allowed us to evaluate what could be transferred from a system to another and how a different species reacts to similar challenges (Box 4). Our studies on five Corsican populations, distant from each other by eight to 105 km, revealed a strong genetic differentiation, with no pattern of isolation by distance (Glémin et al. 2005). More than 10 years of demographic survey on these same populations showed a large variability of population growth among years and populations and a clear tendency of decline (Noel et al. 2010).

### The role of diversity of self‐incompatibility* alleles in the demography of *B. insularis*

The number of S‐alleles at the self‐incompatibility* locus in a population was positively correlated with population size, as expected under genetic drift. Indeed, in the smallest population surveyed, which harbours only 50 reproducing individuals, we found only three distinct S‐alleles, with one of them having a frequency greater than 90% (Glémin et al. 2005). Self‐incompatibility* represents a serious demographic handicap in small populations, but also in larger populations where patch structure may reduce locally the diversity of S‐alleles, and thus the availability of compatible mates (Mignot et al. 2005; Glémin et al. 2008).

### Inbreeding depression in *B. insularis*

In such a situation where the reproductive success of self‐incompatible individuals is strongly limited by the rarity of compatible mates, self‐compatible genotypes could be at an advantage. Evolution towards self‐compatibility would be facilitated by a low inbreeding depression. A small difference in fitness between selfed and outcrossed progeny (which measures inbreeding depression in plants) is expected in small populations, because recessive deleterious mutations of small effect can spread to fixation, while those of strong effect are purged (Glémin et al. 2001). We measured inbreeding depression in controlled conditions, performing crosses with individuals in *ex situ* collections. Contrary to theoretical predictions, we found a large inbreeding depression on survival at different stages of the life cycle (inbred individuals suffered from a 25–30% decrease in survival compared to outbred individuals before first flowering, and a 40–50% decrease after first flowering), which could be explained by the accumulation of mutations during the long life span of the plants (Glémin et al. 2006). Evolution towards self‐compatibility could thus be halted by the large inbreeding depression, despite the constraints associated with the rarity of compatible mates.

### Genetic rescue: risks and benefits

Introduction of individuals with additional S‐alleles in small populations could help improve the reproductive success, by increasing the number of compatible mates. Gene flow could also alleviate the negative genetic consequences of small population size, by restoring genetic diversity at other loci, and making selection against deleterious mutations more efficient. Genetic rescue has been used with success in a few emblematic examples in conservation biology (Hedrick and Fredrickson 2010). Artificial gene flow between isolated populations is however not without risk: isolated populations may have evolved genetic incompatibilities leading to outbreeding depression, and/or have patterns of local adaptation that could be disrupted by gene flow (Tallmon et al. 2004). The five studied Corsican populations of *B. insularis* belong to five different subspecies, raising questions about the genetic entities that are the target of conservation. Would restoring some level of gene flow between populations in *B. insularis* provide demographic benefits that would compensate for the associated risk or loss of genetic originality?

Experimental and theoretical studies provide some insights into this question. On the theoretical side, stimulated by the previous case studies, we investigated the antagonistic effects of gene flow in fragmented populations when both inbreeding and local adaptation affect plant fitness (Lopez et al. 2008). Simulations showed that gene flow had net positive effects in very small populations (with effective size less than 100) because (i) such populations suffer from high drift load and inbreeding depressing their mean fitness, and (ii) local adaptation is generally absent in such small populations as they are too small and genetically depauperate to respond to local selection pressures, consistent with meta‐analyses showing that local adaptation emerges more readily in large populations (Leimu and Fischer 2008). Large populations do not suffer from inbreeding and have higher level of local adaptation, so gene flow is predicted to have less beneficial effects in larger populations. Large numbers of migrants are however necessary to significantly alter the genetic composition of such large populations. Whatever the size of populations, we found that an optimal number of one to two migrants per generation maximized plant fitness, being sufficient to alleviate much of the negative consequences of complete isolation, without severely disrupting local adaptation (Lopez et al. 2008). On the experimental side, in *C. corymbosa* and in the studied Corsican populations of *B. insularis,* we found no evidence for differentiation of functional traits among populations when grown in a common garden, which would be expected in the presence of local adaptation (Petit et al. 2001). These results obtained on the juvenile stage of plants should be confirmed by further analyses of adult traits (because the former are more likely to be affected by maternal effects), and by the study of potential genetic incompatibilities.

## Part 2. Evolution in fire‐prone environments and conservation issues

The two previous endemics seem particularly vulnerable when facing new threats and disturbances, possibly because they have traits adapted to dynamics with very little turnover. In contrast, in the South African fynbos, species are subjected to recurrent natural fires, which burn all above‐ground vegetation and are followed by massive regeneration. Are species adapted to recurrent disturbances more resilient in the context of anthropogenic changes? Our studies of life history traits evolution in the fire‐prone fynbos suggest that the existence of recurrent disturbance in a metapopulation (as opposed to undisturbed habitats) is not sufficient to understand patterns of vulnerability or resilience. Species adapt to specific disturbance regimes, rather than to disturbance *per se*, through different strategies. These specific strategies make some species more resilient or vulnerable than others to changes in the disturbance regime, to climate change, and to other forms of global change.

The metapopulation concept is useful to understand the evolution of life history traits in fire‐prone ecosystems because it sheds light on selective and ecological processes in populations that are far from any demographic equilibrium (Ronce and Olivieri 2004). In the fynbos in particular, the recruitment of many species is conditioned by the occurrence of fire. The age of individuals is then tightly correlated with the time since the last disturbance, which was theoretically shown to select for specific ageing strategies in a metapopulation (Ronce et al. 2005; Cotto et al. 2013, 2014). In addition to seed dispersal, recurrent fires also select for alternative strategies to recolonize burnt sites, such as various types of seed banks (Box 5). The extent of gene flow and dispersal among populations, and their role in the ecological and evolutionary dynamics in the fynbos, are the subject of ongoing investigations. We will here focus on other aspects of metapopulation functioning, those associated with selection pressures in populations subject to recurrent disturbances, which represent both opportunities in terms of recruitment and constraints in terms of completion of the life cycle.

### Conservation issues in the fynbos

The South African fynbos is one of the five existing fire‐prone ecosystems in the world, known for their high levels of endemism and diversity (Myers et al. 2000). Plants from the South African fynbos, and from other fire‐prone ecosystems, display an important diversity in their life history strategies (Rebelo 2001). Such diversity has been attributed to variation in environmental aspects such as soil fertility and components of the fire regime such as fire frequencies, intensities or sizes (Keeley et al. 2011; Keeley 2012), which may have shaped the evolution of these traits in these metapopulations (Box 5). Because of climate warming and human activities, fire frequencies are expected to increase in the future in all fire‐prone ecosystems (Syphard et al. 2009) and could affect the persistence of some life history traits.

### Exaptations versus adaptations: a relevant debate for conservation biology?

The existence of an adaptive link between these life history traits and fire regimes was however challenged by Bradshaw et al. (2011). They proposed that the emergence of serotiny* was not linked to fire, but rather responded to factors such as low nutrient availability and high seed predation. Bradshaw et al. (2011) indeed argued that the emergence of some of these traits predated periods with recurrent fires, and their presence was not restricted to fire‐prone environments. They concluded that the so‐called fire adaptations were most likely ‘exaptations’ – traits that enhance fitness in the presence of a given factor, but which emergence was caused by another factor (Gould and Vrba 1982). Bradshaw et al. (2011) argued that, because some species might be adapted to something else than fire, managing fire regimes may not be the best solution for their conservation.

As suggested by Keeley et al. (2011), an exaptation is a trait that has been shaped by natural selection throughout its entire evolutionary history, and is very likely to be adapted to the conditions experienced currently (see also Gomez‐Gonzalez et al. 2011; Simon and Pennington 2012; Crisp and Cook 2013; Oliver et al. 2013). Keeley et al. (2011) argued that observing that fire‐related traits have evolved multiple times independently (but see Losos 2011), or that they coevolved (as shown by He et al. 2011 in the genus *Banksia*), suggests that fire played a significant role in the selection of such traits. Using phylogenies of *Pinus* and *Proteaceae* respectively, He et al. (2012) and Lamont and He (2012) found that fire‐related traits arose jointly with an important increase of atmospheric O_2_ concentration during the Cretaceous, which probably increased fire frequency on Earth (Bergman et al. 2004). More convincing evidence that fire has shaped the evolution of such traits was provided by models of joint evolution of plant traits and habitat type (i.e. fire‐prone vs nonfire‐prone) along phylogenies, such as was found in the *Proteaceae* family (Lamont and He 2012).

### A different perspective on fire adaptations

In this context, we started to investigate which selective pressures shaped, in the past, the evolution of life history traits in fire‐prone metapopulations and how such knowledge could inform us about potential effects of change in climate and fire regimes. Although understanding what caused the first emergence of a particular life history trait is an interesting question for an evolutionary biologist (e.g. see Bena et al. 1998), it might be irrelevant for conservation purposes. We therefore investigated the more recent evolutionary past of plant groups in fire‐prone environments to understand what is currently maintaining such a large diversity of fire‐related traits, pollination strategies, seed dispersal strategies and degrees of sexual dimorphism (Box 5), combining theoretical studies with comparative analyses.

### Adaptation to fire or to fire regimes? Theoretical considerations

The persistence of serotinous species is supposed to be strongly linked to the distribution of fire intervals. In some fire‐prone environments, recruitment between two fires is supposed to be very rare due to competition with already established plants (Cowling and Lamont 1987; Enright et al. 1998; but see Enright and Goldblum 1999 for an example of interfire recruitment). Thus, serotinous species need to keep their cones closed until fire arrives because their seeds are short‐lived in the soil (Weiss 1984). The degree of serotiny*, defined as the average cone age on the plant, strongly varies among species and among populations of the same species (Cowling and Lamont 1987; Enright and Lamont 1989; Lamont et al. 1991; Midgley 2000; Cramer and Midgley 2009), and is much shorter than the typical mean fire interval, especially in the South African fynbos. Low degrees of serotiny* thus appear as an evolutionary paradox. We used a model of life history evolution in fire‐prone environments, inspired by the biology of the genus *Leucadendron* and other *Proteaceae* in the fynbos, to investigate what the optimal life history of serotinous plants should be under a given fire regime characterized by a distribution of fire return intervals (Tonnabel et al. 2012). We considered that plants allocated resources yearly to growth and survival, seed production, and maintenance of seed canopy. We found that weak levels of serotiny* evolved even in the absence of interfire recruitment. This was because we assumed that resources were limited, generating a trade‐off between current seed production and maintaining past seed production (Tonnabel et al. 2012). We also showed that the variance in fire intervals strongly affects the evolution of the degree of serotiny*: when the predictability about fire intervals is low, the optimal strategy consists in increasing allocation towards current reproduction at the expense of cone maintenance. This is because the optimal strategy is to produce a certain number of seeds every year. This model does not explicitly describe dispersal between burnt and unburnt sites. More recent theoretical investigation showed that low dispersal does not significantly alter the predicted relationships between fire frequency, fire predictability and the evolved life history traits in serotinous species when recruitment between fires is rare (A. Kubisch, J. Tonnabel, F. M. Schurr, and O. Ronce, unpublished manuscript).

### Does the evolutionary history of species inform us about their vulnerability under changing fire regimes?

**Figure 2:**
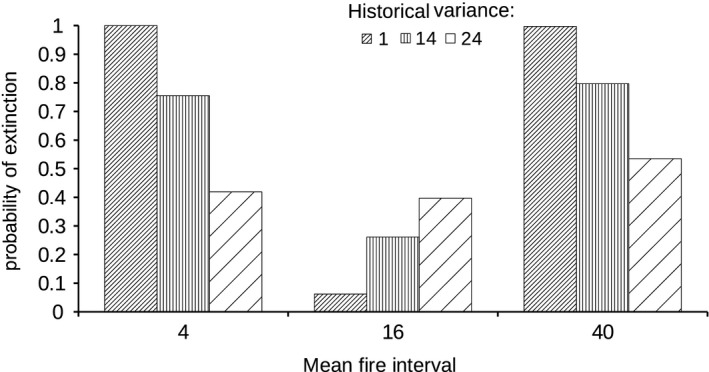
Effect of changes in the mean (*μ*) of the probability distribution of fire intervals (uniform distribution) on the probability of extinction at 50 years for three serotinous populations adapted to different historical fire regimes. For each population, the life history was optimized with a historical mean fire interval of 16 years. Each population had evolved under a given variance of fire intervals. Stochastic simulations were run using the ULM software (Legendre and Clobert 1995).

We used our evolutionary model of life history to predict the demographic consequences of changes in fire regime, assuming that life history traits have been shaped by past historical fire regime. We manipulated both the mean time interval between fires (fire frequency) and the predictability in fire occurrence (the variance of time intervals between fires at a same location). Deviations from the historical mean fire interval always negatively affects population growth and persistence prospects, but populations with larger historical variances are more resilient to a change in mean fire interval (Fig. 2). Whatever the historical variance of fire intervals, a smaller variance in the new fire regime ameliorates the survival of the population if the mean fire interval is unchanged (Tonnabel et al. 2012). Populations with larger historical variances are also more resilient to increased variance. To maintain an adapted life history, one should thus maintain the population historical fire regime. But to maintain biodiversity and *biodiversification*, it is important to maintain a diversity of fire regimes, considering the mean fire interval but also its variance. There are other examples about how knowledge of historical fire regimes can help conservation (see the general review of Dellasala et al. 2004), for instance in mammals (Bilney 2014; Augustine and Derner 2015), birds (Fuhlendorf et al. 2006; Augustine and Derner 2012, 2015), insects (Schlesinger and Novak 2011) and other plants (Menges and Dolan 1998; Marchin et al. 2009; Radies et al. 2009; Weekley et al. 2010).

### Comparative analyses: insights on joint evolution of traits, fire regime and climatic niche

**Figure 3:**
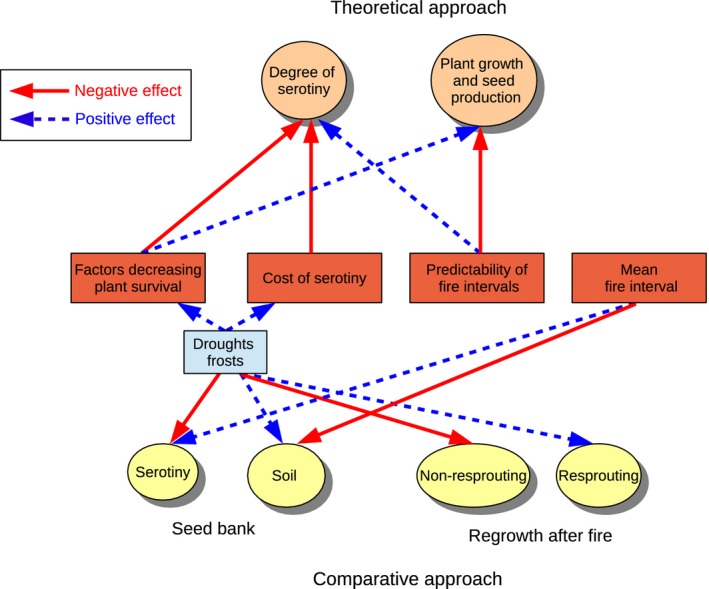
Summary of the results about effects of intrinsic and extrinsic factors on the evolution of life history traits of plants in fire‐prone environments. Dashed blue and continuous red arrows correspond respectively to positive and negative effects. Note that the probabilities of drought and frost were used in the comparative analysis as proxies for factors affecting plant survival and cost of serotiny*.

We tested hypotheses about the adaptive value of different fire‐related traits using macro‐evolutionary comparative analyses. Our goal was to characterize the joint evolution of fire‐related traits and environmental niches to predict how these traits might be affected by potential future changes in climate and fire regimes. We inferred the joint evolution of the environmental niche, for example the mean temperature of the coldest quarter, and that of life history traits along the branches of the phylogeny (J. Tonnabel, F. M. Schurr, F. Boucher, W. Thuiller, J. Renaud, E. M. P. Douzery, and O. Ronce, unpublished manuscript). We expected serotinous species without the ability to resprout after fire to be more sensitive to extreme environments than other trait combinations because their reproduction relies on a single fire event, until which females need to stay alive, and because cone maintenance is costly. We found that the evolution of serotiny* is indeed associated with the evolution of environmental niches characterized by less extreme climatic events, allowing both drought and frost avoidance, and by shorter mean fire intervals (or shorter variance of fire intervals as this aspect of fire regimes is strongly correlated with mean fire interval) compared to species with soil‐stored seed banks* (Fig. 3). The evolution of resprouting* after fire was associated with the evolution of niches with more extreme climates, including more extreme droughts and frosts (Fig. 3). As expected, species combining serotiny* and inability to resprout evolved niches with the lowest exposition to droughts and frosts. We suggest that all combinations of life history traits do not form an even threat in the context of climate change. According to climate change projections, the climate in Southern Africa should become warmer and drier (Tyson et al. 2002). Those serotinous species without resprouting* ability would particularly suffer if summers became warmer, but would benefit from milder winters. Our comparative analyses also showed that life history traits evolved in a correlated manner (Tonnabel et al. 2014): species with greater seed dispersal distances tended to evolve lower pollen dispersal distance, insect‐pollinated species evolved decreased sexual dimorphism compared to wind‐pollinated species, and species with a persistent soil seed bank evolved towards reduced adult resprouting* ability after fire compared to serotinous species. This suggests that environmental changes would probably threaten suites of traits rather than single traits.

## Part 3. Implications for management

### Halting decline and founding new populations in *Centaurea corymbosa*

The Clape Massif, where all populations of *C. corymbosa* are found, belongs to the European network of nature protection areas Natura 2000. The management plan for this site in the context of Natura 2000 was written jointly by researchers, experts (National Botanical Mediterranean Conservatory of Porquerolles CBNMP) and managers (Narbonnaise in Méditerranée Natural regional Park, Coastal Protection Agency, Forests National Office, Gruissan City). The demographic survey in *C. corymbosa* shows a clear decline of populations, with a high risk of extinction within 50 years. Population decline is linked with the progressive encroachment of dense vegetation on the habitat of the poor competitor *C. corymbosa*, at the edges of cliffs, due to the abandonment of agricultural practices, the decline in sheep grazing, and fire control. Management should therefore aim at actions favouring more open habitat, through, for example, controlled clearings, to first halt the decline of extant populations. More open habitat on the plateau could also help connecting different patches of favourable habitats, allowing the colonization of empty patches from occupied patches. Colonization of new patches of habitat would in turn result in different selection pressures on life history traits and mating system, allowing the maintenance of greater diversity in the populations, slowing the downward evolutionary spiral in which this species seems to be caught.

A second line of action concerns the new populations created in 1994 and 1995: one which just maintained itself with 67 individuals in 2014, while the other one went extinct in 2014. Reinforcement or reintroduction of large numbers of seeds would be necessary to overcome Allee effects, but would require a phase of multiplication and seed production in controlled conditions to avoid collecting too many seeds *in situ* in already declining populations. A protocol for the reintroduction programme has been elaborated jointly with managers, and plans for its funding are under discussion. Demographic reinforcement may increase the probability of successful establishment of these new populations, but will diminish the strength of selection on genotypes with alternative mating and life history tactics during such artificial colonization.

### Reinforcement of populations in *Brassica insularis*

Our demographic surveys in *B. insularis* allowed us to identify specific threats (or the absence of immediate threats) acting on the different surveyed populations, which include disturbances by climbers, overgrazing, herbivore attacks and fires (Noel et al. 2010). The National Botanical Conservatory of Corsica has in particular implemented a communication campaign towards users of the sites to reduce those threats. In the very small populations, these direct demographic threats may be aggravated by the lack of compatible mates, due to the loss of genetic diversity at the S locus. Reinforcement of populations has been proposed in management plans of the species. Our single attempt to introduce a new population in an empty site has failed, due to unfavourable weather, but reinforcement of extant populations through the establishment of additional individuals has been successful (unpublished data). How should we choose the genetic origin of the plants used for reinforcement of extant populations? There exists an *ex situ* conservation scheme in *B. insularis* consisting in a seed collection of diverse origins managed by the CBNMP. Because genetic diversity at the S locus may be instrumental for the positive effect of such reinforcement, it would be interesting to evaluate how the collection maintains this diversity both within and between populations. When local diversity has been lost both *in situ* and *ex situ*, the use of nonlocal material should be discussed, balancing the costs and benefits of such interventions.

### Consequences of fire management in the genus *Leucadendron*

The South African Cape Floristic Region (CFR) is a priority for conservation (Myers et al. 2000), in which both national and provincial conservation organizations (i.e. SANParks and CapeNature) have invested great efforts. CapeNature, a provincial public entity of the Western Cape Government, is a key conservation unit responsible for managing and monitoring fires in protected areas of the CFR. Conservation policies are thus greatly affecting fire regimes. In the South African fynbos, fire suppression was adopted at the beginning of the 20th century and later abandoned for a management strategy called prescribed burning, which consists in burning patches of land with a fixed mean fire interval (van Wilgen et al. 2010). These management strategies ignore the fact that many organisms and their life history strategies might be adapted to specific fire regimes that they experienced in the past. Our studies on *Leucadendron* suggest that information about historical fire regimes should be incorporated into prescribed burning strategies when they are implemented. Serotinous species were found to have evolved jointly with shorter mean fire intervals or shorter variances of fire intervals, which may make them particularly sensitive to management practices decreasing frequencies or predictability of fires. So far, prescribed burning practices lack a scientific method when choosing an artificial mean fire interval (but see Kraaij et al. 2013). Managers and scientists should work together to build an integrative method for prescribed burning strategies while accounting for past fire regimes, site specific diversity of life history traits, and potential threats to human populations.

Overall, our results, both theoretical and empirical, suggest that a diversity of environments and disturbance regimes have selected for a diversity of life history traits and strategies adapted to these different conditions. Thus, it is crucial to maintain a diversity of environments in the fynbos to maintain the extraordinary diversity of life history traits in this ecosystem. Uniform fire regimes through prescribed burning with similar artificial mean fire intervals may allow the maintenance of only those species which by chance evolved under similar regimes, or those adapted to very variable fire intervals, which we predict to be more resilient to change in fire regimes.

## Conclusion

**Figure 4:**
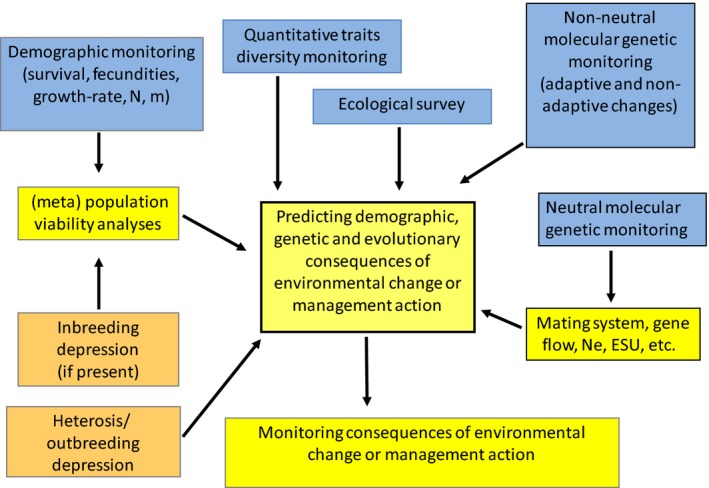
Planning research in Evolutionary conservation biology. Blue boxes correspond to demographic, ecological and genetic surveys (i.e. monitoring over several years), yellow boxes correspond to calculations, and orange boxes correspond to experiments. Arrows indicate that the box it is starting from should be taken into account in the box towards which it is pointing.

We think that conservation of biodiversity should aim at conserving evolutionary processes that generate biodiversity. To maintain processes leading to biodiversity, it is essential to maintain a diversity of landscapes. The case studies presented in this study illustrate how we identify the threats on biodiversity at different scales of organization, integrate eco‐evolutionary feedbacks in our evaluation of risks, propose new indices of vulnerability and imagine different management practices. The application of genetics in the management of threatened species in the wild is still in its infancy (Laikre et al. 2009). According to Frankham (2010), this is not due to a lack of scientific guidelines, but to the failure to consider genetic issues in wild management (Fig. 4).

Integrating evolutionary considerations in conservation practices is not an easy task. Defining a research programme for evolutionary conservation biology requires integrating many different complementary approaches, including nonevolutionary ones (see attempts to define such an ideal programme in Fig. 4). Adaptive evolution is mainly concerned with the diversity of life history traits (Hansen et al. 2012). How should we monitor genetic diversity for adaptive traits? In the optimal situation, one can follow a gene (see examples from pesticide resistance: Weill 2013; McNair 2015). But usually, there is no candidate gene: monitoring phenotypic diversity is then necessary. Controlled conditions experiments should be set up whenever possible to measure quantitative genetic variation for adaptive traits both within and between populations (Petit et al. 2001). Genetic monitoring (surveys through time) should be done on quantitative traits as well as on neutral and non‐neutral genes in several populations. Neutral genes will inform us on evolutionarily significant units, mating systems and gene flow. Quantitative genetics and non‐neutral genes will inform us on adaptive diversity. Genomics offer new powerful tools for genetic monitoring. Demographic survey (i.e. identifying and counting the various ages or stages at every *census*, on several occasions, as we did on *C. corymbosa* and *B. insularis*) will allow us to perform viability analyses, complemented by data from crosses on inbreeding and outbreeding depressions. Together with ecological survey (i.e. following the habitat), demographic and genetic surveys (i.e. following genes) will allow us predicting the consequences of management actions. These predictions then need to be tested.

Through our studies on *C. corymbosa*,* B. insularis* and the genus *Leucadendron*, we explored several facets of these complex interactions and feedbacks between genetics, evolution and ecology: each facet enriched considerably our understanding of the functioning of threatened species and ecosystems. This was an exciting experience, and we hope that many will embark on such a research programme with great both scientific benefits and benefits for the protection of biodiversity.
